# THE ASSOCIATION BETWEEN LOWER EXTREMITY FUNCTION, FRAILTY, AND LOW-MILEAGE DRIVER STATUS AMONG OLDER ADULTS

**DOI:** 10.1093/geroni/igz038.2528

**Published:** 2019-11-08

**Authors:** Christopher L Crowe, Howard Andrews, Lisa J Molnar, David W Eby, Carolyn DiGuiseppi, David Strogatz, Guohua Li, Thelma J Mielenz

**Affiliations:** 1 Columbia University Mailman School of Public Health, New York, New York, United States; 2 University of Michigan Transportation Research Institute, Ann Arbor, Michigan, United States; 3 University of Michigan Transportation Research Institute and Center for Advancing Transportation Leadership and Safety, Ann Arbor, Michigan, United States; 4 Colorado School of Public Health, Aurora, Colorado, United States; 5 Bassett Healthcare Network, Cooperstown, New York, United States

## Abstract

The crash rate per mile driven among older adults is higher than that of most age groups and comparable to that of the youngest, most inexperienced drivers. The low-mileage bias posits that the elevated rate among older adults results from an increased rate among those who accrue the fewest annual miles. This study evaluated whether low physical capacity among older drivers, measured by the National Health and Aging Trends Study (NHATS) Expanded Short Physical Performance Battery (SPPB) and Fried’s frailty phenotype, increases the risk of being low-mileage drivers. Data were collected for 2,990 older drivers via questionnaires and assessments in addition to 61,528 person-months of driving data. Multivariable log-binomial regression was used to estimate risk ratios. Those with fair and good function had 0.53 (95% CI: 0.40-0.69) and 0.60 (0.47-0.78) times the risk of driving fewer than 3,000 miles/year and 0.45 (0.26-0.77) and 0.48 (0.32-0.72) times the risk of driving fewer than 1,865 miles/year, respectively, compared to those with poor function. For an increase from not frail to pre-frail and from pre-frail to frail, the risk of driving fewer than 3,000 or 1,865 miles/year increased 1.36 (1.11-1.65) or 2.38 (1.63-3.46) times, respectively. Having low physical capacity is associated with an increased risk of low annual mileage. Given the known association between low-mileage driver status and increased crash rates and the modifiable nature of the risk factors examined in this study, interventions aimed at improving physical capabilities may lead to an improvement in safety among older drivers.

